# The Hunt for Kinder Practices: Minimising Harm to Wild Boar Welfare, Insights from a Qualitative Study in Wallonia (Belgium)

**DOI:** 10.3390/ani14233370

**Published:** 2024-11-22

**Authors:** Pauline Emond, Dorothée Denayer

**Affiliations:** Équipe SEED (Socio-Écologie, Enquête et Délibération), UR SPHERES, Département des Sciences et Gestion de l’Environnement, Faculté des Sciences, Université de Liège, Avenue de Longwy, 185, 6700 Arlon, Belgium; d.denayer@uliege.be

**Keywords:** hunting, wild boar, wildlife welfare, qualitative survey, sociological method, one health

## Abstract

As in the rest of Europe, the Belgian boar population has been tending to increase, posing new challenges to management by hunting. Moreover, in autumn 2018, the African Swine Fever (ASF) virus penetrated Belgian territory. In response to this so-called overpopulation and to this health crisis, wild boars were massively culled by hunters and other nature managers. The wild boar was then debated by some stakeholders as a sensitive animal and that its welfare mattered even if it must still be killed. In this article, we explore the management of wild boar through a qualitative sociological survey focusing on damage to the welfare of wild animals and ways of limiting it. This survey is part of an innovative process launched by the Walloon Region and the Walloon Council for Animal Welfare (CWBEA). It was conducted in order to qualitatively describe current hunting practices and their impact on animal welfare; it then integrated knowledge from the human sciences into the CWBEA’s work; and, finally, those from the hunting world were invited to debate with the usual animal welfare advocates and managers. By identifying, based on the knowledge of hunting stakeholders and wildlife specialists, a multitude of issues and avenues for action to limit the harm to the welfare of wild boars, this paper highlights the relevance of an interdisciplinary and transdisciplinary approach to the welfare issues of wild animals. It supports the hypothesis that animal welfare advocates can work toward establishing new norms in human/nonhuman relationships in collaboration with hunters but also to include wild animals—wild boars in this case—as sentient beings whose welfare must be considered and debated in discussions on global health.

## Simple Summary

As in the rest of Europe, the Belgian boar population has been tending to increase even with the African Swine Fever prevalence, posing new challenges in their management by hunting. Indeed, wild boars have been massively culled by hunters and other nature managers, but some stakeholders argued that they are sensitive animals and that their welfare matters. In this article, we explore the management of wild boar hunting through a qualitative sociological survey focusing on damage to the welfare of wild animals and ways of limiting it. By identifying, based on the knowledge of hunting stakeholders, a multitude of issues and avenues for action to limit the harm to the welfare of wild boars, this paper highlights the relevance of an interdisciplinary and transdisciplinary approach to the welfare issues of wild animals. This study advocates including wild animals—wild boars, in this case—as sentient beings whose welfare must be considered and debated in discussions on global health.

## 1. Introduction

Like in the rest of Europe [1], Belgium’s boar population has been on the rise for many years [2,3], creating challenges for its management through hunting. Moreover, in the autumn of 2018, capitalising on the high density of wild boars, the African Swine Fever (ASF) virus spread to Belgian territory, leading to the implementation of extensive measures to cull the animals [4]. These included targeted wild boar hunts, night shooting by trained forest officers, and trapping followed by killing. Hunters were enlisted, among other nature managers, for their hunting skills to carry out a part of these eradication operations. As a result, the perception of wild boars shifted from being valued game to a massive health threat and a pest animal targeted for elimination. In the health management proposed by experts, the short-term health of domestic animals was considered a priority and was completely decoupled from the issue of the health and welfare of the wild boars. On the ground during culling operations and especially during trapping, some trappers, who were usually hunters or forest officers, raised concerns about the impact on wild boar welfare. More widely, these new practices, which involved capture followed by killing, highlighted welfare issues within this new community of trappers; they were questioning the stress, distress, and physical suffering that the wild boars experienced during these processes. As culling operations progressed, the trapping by some trappers and stakeholders evolved by incorporating these considerations. For example, they implemented a type of trap that causes fewer injuries, the circular trap, due to the absence of angles. They also recommended placing opaque screens around the traps to minimise stress. The administration asked prospective trappers to accept a code of conduct to ensure respect for certain animal welfare concerns. Even some sedation tests had been conducted by a trapper and the administration to try to calm the boar while they were trapped. A report on trapping [5] was produced by the administration, including a section on welfare that incorporates these recommendations. Alongside this concern for welfare, these operations paradoxically revealed the skills of wild boars in such interaction with humans [6]. Wildlife managers thus positioned themselves as spokespersons for an issue that has so far been overlooked: the welfare of wild animals destined to be killed.

This continued and even intensified afterward, influenced by the input of civil society actors. A collective led by six environmental NGOs, and supported by 64 others, created in 2019 [7] was pressuring the Walloon government and called for a debate on the social, environmental, and ethical consequences of what they identified as “hunting abuses in Wallonia”. This collective denounced the very existence of game animals and their advocacy framed the existence of wild boars as sensitive creatures that needed to be killed to maintain population control and reduce health risks. The welfare of wild animals was seen by some of these stakeholders as a challenge to hunting itself.

Even so, at the end of 2020, Belgium regained its status regarding ASF-free wildlife at the European and worldwide levels [8,9]. On 10 May 2021, Céline Tellier, the Walloon Minister for Animal Welfare, requested that the Walloon Council for Animal Welfare (CWBA) (a government body is usually responsible for the welfare of domestic and livestock animals) draft a report with recommendations to minimise harm to animal welfare in hunting practices and the culling of nuisance animals, with exceptions for the destruction of protected animals. These recommendations covered hunting methods, wildlife management, trapping, as well as practices for killing and releasing game in recreational hunting. In early 2022, the SPW Agriculture, Natural Resources, and Environment launched a public tender to find an expert to assist the CWBEA in preparing a report [10] for discussions on hunting and producing an opinion. The SEED team from Liège University, specifically the two authors of this article, won the tender for the preparation of the report providing a methodological proposal and data collected on Walloon hunting since the 2019 ASF crisis as part of a doctoral thesis. This report, based on a field survey, is part of an innovative process launched by the Walloon region to first put the CWBEA to work on wild animals; second, to integrate knowledge from human sciences into the CWBEA’s work; and, finally, to invite players from the hunting world to debate with the usual animal welfare advocates and managers (The animals and practices considered in this work were initially all wild animals killed through hunting, culling, or regulation. Given the scope of the task, this work had to be divided by focusing, first of all, on a subset of these animals, specifically big game and hunting practices. We decided in this article to focus solely on wild boars and their relationship with hunters, although the results are mostly applicable to other large game species, as they were produced with this specific focus in mind.). When this report was finalised in 2022, it was presented to the members of the CWBEA. Then, they formed a working group of about ten people, consisting of CWBEA members who had expressed interest in participating, as well as external individuals, some of whom were encountered during the preliminary survey. Special attention was given to the composition of the group to ensure representation of the various stakeholders involved and the diversity of viewpoints. Thematic guests participated in meetings according to their expertise and the theme of the meeting. This group convened five times for half a day during the year 2023 and established an initial list of recommendations approved in 2024, but this work is still ongoing. Here, we focus on the exploratory investigation that served as a basis for this group’s work. This investigation thus covered a broader scope, including certain aspects that have not yet been addressed or not yet within this working group.

For this article, the theoretical framework of the One Health approach was useful, as it advocates for greater consideration of animal welfare in research and decision making related to global health. In the One Health perspective, animal welfare research is “part of a wider endeavour to optimise the health and well-being of humans, animals and ecosystems” [11]. The World Organization for Animal Health (WOAH) defines animal welfare as “the physical and mental stage of an animal in relation to the conditions in which it lives and dies” [12]. In the context of health crises or pandemics, animal welfare should be taken into account in decision making [13]. The literature usually defines respect for animal welfare as first taking into account the animal’s condition as a sentient being (capable of physical and psychological suffering) and second using subjectivity (intentionality, emotions) to consider the animal’s needs as determined by its physiology and specific place in the natural environment [14]. Therefore, the assessment of animal welfare involves a combination of approaches, fields, and disciplines. Animal welfare can be partially understood through recent scientific knowledge related to the animal and its physiology but can also be more precisely evaluated using complementary types of measures, such as zootechnical, semiological, physiological, and ethological assessments [15]. But, animal welfare is not only a concern for the animals themselves [16] or for scientists but also for the many who are ethically concerned by this welfare in their practices and decisions [17]. So, animal welfare research methods should aim to include a diversity of voices and knowledge [18].

Our exploratory analysis of the scientific literature revealed a significant gap between the abundant scientific studies on the welfare of domestic animals (in the broad sense) and the much less but emerging literature dedicated to the welfare of wild animals [19], even though some tried to tackle both as common goals [20]. Generally, the study of welfare of animals directly involved in human activities (laboratory, farm, or zoo animals) has often used wild animals as a reference to identify the needs of those in captivity. Nevertheless, the distress and well-being of wild animals are often linked to direct or indirect anthropogenic disturbances [21,22] like habitat degradation [23], climate change [24], or tourism [25], and this situation tends to intensify in the context of an ecological crisis. Reducing the impacts on the welfare of wild animals resulting from anthropogenic practices does not mean ensuring a life of well-being for wild animals. Reflecting on the total and absolute well-being of each animal thus makes little sense because this goal escapes our control due to the very nature of wild animals. However, during their killing by our hands, the human, as an empathetic predator [26], has control over their suffering and can ask how to alleviate it.

Many works in the social sciences have analysed hunting around the world from different aspects and perspectives, such as the philosophical and existential approach to hunting [27] or by analysing its ritual aspects, for example, in France [28,29]. Other authors, like Bertrand Hell, have been interested in the myths related to hunting and the wild human [30,31]. Tim Ingold provided an approach focused on the environment and how the hunter perceives it [32], while Roberte Hamayon studied hunting in Siberian animist cultures as a form of connection and relationship [33]. In 2005, Descola offered a reflection on identification regimes and the differences between animism and naturalism in hunting [34]. Others examined hunters’ perceptions of animals and the inherent violence of hunting [35], analysed it in terms of values [36], or focused on its processes of “ecologization” [37]. Some adopted a socio-technical and meta-technical approach, like Govoroff, with firearms and ammunition, which are inherent in most modern hunting practices [38]. Historical anthropology studies of hunting have also been conducted [39]. More recently, Charles Stepanoff described the crisis of the wild and hunting in modern France, highlighting dualisms and conflicting relationships with nature and wild animals [26].

More precisely, regarding the consideration of welfare, several authors have raised general issues, such as Scruton [40] and Winkelmayer [41], who discussed ethical perspectives and welfare in hunting. Meanwhile, others have taken a more specific approach, such as addressing underaddressed animal welfare issues in recreational hunting in Australia [42]. Other authors approached these themes through the perspective of hunters, like Mounet and Chanteloup [43], who studied how hunters navigate societal injunctions regarding animal welfare and sensitivity, as well as how they perceive death and animal sensitivity within their practices. Von Essen [44] examined hunters’ use of care language in her article. One author analysed the normative approach and perspectives in the current animal welfare law regarding hunting [45]. Some studies attempted to analyse impacts on welfare with an approach more centred on practices or animals, such as the welfare assessment of the methods used for the harvesting, hunting, and population control of kangaroos and wallabies [46] or the hunting of captive-bred lions in enclosures [47]. The welfare of pig-hunting dogs in Australia was also questioned [48], as well as the traditional Sami hunting of ducks in spring [49]. Others tried to quantify this more precisely and define animal welfare standards, such as how body mass determines thresholds for incapacitation time and flight distance during hunting [50] or by testing recommendations for shooting and darting [51]. A study measured cortisol levels (the stress hormone) in wild boars killed during drives to analyse the effect of hunting methods on animals [52]. In Belgium, studies on this subject are lacking. Hunting in Belgium is poorly studied and understood. Often, hunting is addressed on the fringes of other more “strategic” topics for researchers like forest history [53] environmental management [54], African Swine Fever crisis management [55], or the impacts of shooting quotas on hunters [56].

In this study, we pursued several objectives. First of all, we aimed to qualitatively investigate the current hunting practices and their impact on animal welfare. Our hypothesis was that it is not possible to limit the impacts on animal welfare without understanding the practices involved, such as understanding hunting in this context. Our goal in this article was to link welfare to the actions of managers, rather than to an objective reality inherent to the animal. Therefore, we analysed the managers’ perspectives on welfare, which are obviously connected to their views on their own practices and those of other hunters. The originality here lies in being a socio-anthropological investigation, that is, producing useful knowledge to achieve this objective by relying on the knowledge and skills of the actors involved, directly or indirectly, in the practices that impact the welfare of wild animals. Then, we aimed to include field contributors in the knowledge production. Indeed, welfare serves as a context and catalyst, reshaping the way we think about hunting and linking it to research, administration, and animal protection. In this work, we drew on and utilised the concept of welfare as an object of qualitative inquiry, with the aim of seeking to minimise harm, through the perspectives of the actors responsible for such harm. Finally, and more broadly, we aimed to integrate animal welfare issues into an interdisciplinary and transdisciplinary approach, aligning with the One Health framework that encourages the blending of ecological, health, veterinary, and social perspectives.

The main hypothesis in this study was that wild animal welfare is significantly influenced by human decisions, relationships, and systems regarding their killing. It highlights how human practices shape animals’ lives, focusing on the harm they experience. This study aimed to understand this harm and analyse the technical, sociological, and legal mechanisms causing it. Our project therefore aimed to contribute with social science and to highlight the value of qualitative research and its framework on these issues. In this article, we focus on our subject, the wild boar, which currently benefits from abundant but very sector-specific literature. As Erika Von Essen highlighted in her article [57], the wild boar is generally studied from the perspective of its management methods. While she concentrated on the impact of the wild boar on hunting and its communities of hunters in terms of activating new or old values, identities, and norms, our aim here was different: to account for how the actors involved in the hunting and killing of wild boars would modify their practices to limit the harm to the welfare of the animals they kill. Moreover, wild boar is fascinating to think about because it encompasses several worlds. As we can see here, it involves not only the world of hunting but also that of management, environmentalists, and animal protection. What is intriguing is how the wild boar brings all these worlds to work together and how each of these worlds provides a partial and biased understanding of it.

In Wallonia, wild boars holds an important place in hunting dynamics and is the most-hunted big game species. They can currently be killed throughout the year. In the context of hunting, their killing by stalking or stand hunting is permitted year-round, both in open fields and in forests. However, driven hunts and hunting with hounds are subject to specific periods: they are allowed in open fields from 1 August to the last day of February and in forests only from 1 October to 31 December inclusive for the 2024–2025 hunting season [58]. Additionally, in the context of control measures, wild boars can also be eliminated year-round using traps, night shooting in open fields, or extra-driven hunts. These actions, however, require adherence to specific conditions and the acquisition of the necessary authorisations [59].

## 2. Materials and Methods

The materials for this investigation were produced in two distinct stages. First, a minimal part of the data were obtained from a survey conducted since 2019 as part of a four-year research project (2020–2024) funded by the National Fund for Scientific Research (FNRS) of Belgium on hunting, wild boar, and the ASF crisis. Then, the majority were gathered during the preliminary investigation for this report [10], conducted between August and November 2022 throughout the Walloon territory. This report was intended to serve as a working basis for the group assembled by the CWBEA afterward and to provide an initial exploration of this issue.

The preliminary survey for this report was conducted with a qualitative and socio-anthropological field investigation and an inductive approach. By focusing on the experiences, knowledge, and projects of those involved in the culling of wild animals in the Walloon region, emphasising their humane treatment rather than their direct well-being. Indeed, we were not directly investigating the animals themselves regarding their welfare; we were investigating their treatment in practice. This means that we relied on the testimonies of the relevant actors to try to understand, together with them, the full complexity of this issue. The aim was to identify the relationships between hunting activities, culling, and the welfare of wild animals, as well as to propose detailed and realistic qualitative reflections for improving their welfare. The question we posed to hunters here was not “how to ensure the welfare of wild boars” but rather “how to minimise harm to their welfare in your practices”.

In qualitative research, it is less about investigating the occurrence of social phenomena and more about understanding the nature of these phenomena and how actors describe and associate them. Qualitative approaches thus make it possible to reveal aspects of a problem that are not directly visible through a quantitative approach: the diversity of practices, actors’ strategies, and the variety of viewpoints. They are particularly well suited for situations that require identifying new opportunities or when the researcher is confronted with a “social reality to be discovered” [60].

In our investigation, we were engaged with a variety of stakeholders to understand not only the current situation but also potential transformations in favour of animal welfare. The results of this initial survey revealed a large panel of issues. The perspectives gathered were diverse, with varying degrees of disagreement among the actors, and, sometimes, their views were in complete opposition. The lack of consensus should not be seen as a waste of time or a failure but rather as a constructive element of the debate. Public controversies have real social productivity, both as a way of exploring issues and as a means of learning about the scenarios and arguments surrounding them [61]. In other words, the disagreements between actors reveal the consensus on the issues that matter. The issues are thus defined in the form of questions, problems, and concerns around which the participants converged, even if they may have different viewpoints and do not rely on the same experiences and observations. Working in terms of issues was also simply a method for transforming complex qualitative data woven together into something workable with the group. Indeed, following this report, a working group composed of veterinarians, ethologists, hunters, ecologists, and members of animal protection associations was established to explore the results and reach a consensus on a list of recommendations to be submitted to the minister. Due to our preparatory field survey, we were also invited to participate in this working group, not as stakeholders, but as sociologists and scientific experts.

The preparation of the fieldwork involved identifying and selecting the contributors to the interview based on a general mapping of the issue. Following this, 22 resource actors were interviewed. They ae referred to throughout this article by the terms ‘actors’, ‘stakeholders’, ‘participants’, ‘contributors’, or ‘interviewees’, and a detailed description of their status, profiles, selection, and methods of interviewing is provided in Table 1. The selection of individuals should be understood here as the process by which these names appeared on our potential interview list. Based on the actor mapping conducted earlier and this list, we then selected some participants. As we collected data and received recommendations from others during the interviews, we expanded the list and met with more contributors. When we decided that we had gathered enough diverse perspectives and data for the scope of our investigation, we stopped the interviews. Particular attention was paid to the diversity and competence of the people interviewed, balancing practitioners and institutional representatives. The anonymity of the actors was ensured to allow them to speak freely on this sensitive topic.

Focusing on actors’ practice narratives [62], we conducted semistructured interviews, which were open discussions guided by an interview guide (the standard one can be found in the Supplementary Materials as a supplementary file). This guide was adapted for each interviewee while ensuring question comparability to produce standardised results [63]. Some contributors requested to receive the questions beforehand to better prepare or provide collective responses. Some interviews were recorded to obtain the most accurate data possible and to closely adhere to the participants’ statements during the subsequent analysis stages. However, some individuals refused to be recorded. Nonetheless, all interviews were documented with notes.

We then analysed these data using the CATWOE framework [64], derived from Soft System Methodology [65,66,67]. This analytical tool, originating from management sciences, allows for a detailed understanding of the gaps and convergences between the logic of different actors or groups of actors, which is a first step towards identifying areas for manoeuvring to find solutions: What are the world views of the different interviewees? What changes are desired? Who would be the victims or beneficiaries? The analysis grid is presented in Table 2.

The definition of the problem by the actor relates to the elements E, W, and O; T and A refer to management strategies; and, finally, C refers to the consequences for the concerned parties.

This grid was used to break down the speeches of each stakeholder interviewed. It provided a structured layout of the raw data. This layout then allowed for a symmetrical analysis of the viewpoints and their comparison in order to highlight shared issues despite the diversity of stakeholders. The results of this comparison that are presented in the “Results” Section of this article, and not the grids themselves. This identification of points of divergence and convergence (synergies) among the various participants could serve as a basis for designing future debates. It is an approach that aims for, and ultimately enables, social consultation. The objective of this stage was to make the debate around complex issues clear and understandable in order to encourage the different participants to support potential transformations of the situation [68].

The interviews were complemented with field data, like ethnographic observations and the grey literature, such as statistical data from the associations or from the administration, but also, for example, the review book for preparing for the hunting exam. During previous missions, we conducted immersion and participant observation [69] on various wildlife culling grounds, including different types of hunting, trapping for monitoring, and culling wild boars. The 172-page report [10] was finalised at the end of 2022 and submitted to all members of the CWBEA for a presentation meeting of its conclusions by the two authors. These results were then discussed in the working group specifically formed to develop a list of recommendations to provide to the minister. Although an initial list of recommendations was established by consensus among the members during meetings where the debates were confidential, their work is still ongoing, and the results of this work are therefore not presented in this paper. Some clarifications and data already produced by this group, published in their list of recommendations, are provided sometimes for additional precision, and the complete list of recommendations can be found on the CWBEA website in the published notices [70].

In the Results Section of this article, you can also find descriptions of the hunting practices currently in use in Wallonia. These descriptive details are an integral part of the results of our investigation, as they were developed through interviews and the documentation provided by the participants. The starting point of this investigation was describing the hunting practices in the Walloon region, as they have rarely been documented, making it necessary to detail them through this study.

## 3. Results

### 3.1. Issues Related to Hunting Practices

According to the testimonies of our interviewees, significant differences exist between the various methods of hunting wild boar used in the Walloon region. These disparities entail specific risks to animal welfare, which were extensively discussed by the actors we interviewed. These different hunting practices depend on the biotopes, territory size, management needs, season, as well as habits of the hunter and of the boar. Hunting methods are also linked to the cultural and traditional specifics of a region. While the hunter–wild boar relationship is certainly central, it is directly impacted by the expertise of other stakeholders, such as scientists, associations, and the administration.

#### 3.1.1. Different Hunting Practices in the Walloon Territory

Driven hunts (“à cor et à cri”), which are collective, loud, and sociable, represent the most common form of hunting wild boar in Wallonia, both in terms of the number of animals killed and the participants involved. This means that many animals are affected. The hunting conditions during driven hunts with horns and shouting generate greater risks regarding animal welfare. Hunters, positioned in line, shoot at game d systematically riven towards them by beaters, with the use of dogs and loud noise-making instruments. The beaters advance in a line, driving the game towards the stationed hunters, who can only shoot when their target cross the line and at an angle greater than 30° from the line for safety reasons. Dogs play a crucial role in flushing out the game. If a wild boar is injured or caught by the dogs before crossing the hunter’s line, the beaters kill it with blades to minimise suffering and avoid injuring the dogs. Hunters have to kill injured wild boar but most often only after the drive for safety reasons. Most of the interviewees pointed to the driven hunt as a source of stress for the animals. In these hunts, wild boar are pursued and intentionally frightened to induce a flight response, aiming to shoot them while they are running. Regarding whether the stress induced is at an abnormal level, a part of our participants noted that in the absence of predators, such practices maintain the flight behaviours of animals that are naturally prey. For these stakeholders, maintaining this behaviour is justified, as a fear of humans would also help limit wildlife intrusions into human-occupied areas. Others lamented that these behaviours make animals much harder to approach and observe. Beyond the possible stress caused by driven hunts, some of our actors told us that the injuries resulting from these hunts are too numerous and proportionally more significant compared to practices involving shooting stationary wild boar. The higher proportion of injuries in driven hunts could be specifically related to the principle of shooting moving animals, according to them. However, the common factors related to the behaviour and the specificities of targeted animals, as well as the actual skills of hunters and the effectiveness of firearms and hunting equipment, were also mentioned. These factors are not unique to driven hunts and were addressed further when analysing cross-cutting issues.

Tracking hunting blinds (traque–affût) or silent push/drive is another type of collective driven hunt, inspired by German practices. Unlike classic driven hunts, hunters are positioned on high stands within the hunting area and can shoot 360°. Beaters advance quietly without shouting or using horns, sometimes with few or no dogs. This is why it is sometimes called a silent drive, even though the use of this term is discouraged by some contributors. High stands must be stable and well placed for accurate shooting. Rules include shooting only at stationary game, except the wild boar, which can be shot on the move within 50 m. The forest does not need to be closed, and beaters wear fluorescent clothing. This hunting method is challenging in dense biotopes and is often practised late in the season. For a significant part of the interviewed contributors, the debate on the future of hunting and wild animal welfare concerns potential alternatives to traditional driven hunts. Among the experimented alternatives are silent pushes or stalking. The major differences are that the boar are shot with less haste, when moving more slowly, with a better shooting angle, in a more effective shooting position for the hunters, and with optics that can be more precise.

Hunting from a hide or ambushing and stalking are individual hunting methods. Ambushing involves waiting for a game from a fixed position, often elevated. Stalking, or “pirsch,” requires the hunter to approach the game undetected. These practices occur mainly at dawn or dusk and require good knowledge of the terrain and the wild boar’s habits. The goal is a precise, fatal shot, often used for selective shooting like culling sick animals. Hunting by individual methods like approaching and or waiting was pointed out by most contributors as the method that causes the fewest injuries due to more favourable shooting conditions.

Usually, hunters stated that each hunting method has its strengths and weaknesses. The driven hunt with dogs and horns would be effective at flushing game and is very sociable, but it could cause more injuries and stress to animals according to the statements of some of the stakeholders we met. The tracking hunting blinds hunt is quieter, allows for more precise shots, and limits forest disturbance, though it is more demanding to implement. Stalking is complementary, allowing clean shots of stationary animals and low-moving wild boar but may not suffice to meet hunting quotas. Some contributors promoted these alternatives (tracking hunting blinds hunt and stalking/ambushing) to driven hunts, while others expressed reservations that need to be taken seriously to advance the broader deployment of these hunting methods. These reservations included (1) the suitability of these practices for different territories, (2) the ineffectiveness of these practices for certain animals, particularly wild boars, (3) the limited role of these practices in regulating wild boar population density, (4) the impact of these practices on wild animal behaviour, (5) the actual impact of these practices on animal stress, and (6) the lack of statistical data to objectively assess the effectiveness of these practices in reducing injuries.

The effectiveness of each method mostly depends on the environment, and hunters often specialise in a particular method. Historically, driven hunts are more common in Latin tradition countries and are considered “gathering” hunts. In contrast, stalking or stand hunting, practised individually, is more common in Germanic regions and considered “harvesting” hunts. In Wallonia, both practices coexist, but driven hunts, especially with dogs, are the main hunting method, particularly in the extreme south. Conversely, in the Eastern Cantons along the German border, stalking and ambushing are preferred, combined with collective tracking hunting blinds.

#### 3.1.2. Bow Hunting

Bow hunting is not yet regulated in Belgium; thus, it is neither forbidden nor officially permitted. This previously marginal practice is growing. Most archers hunt use the stalking method but also participate in drives for stationary or slow-moving animals. However, new hunting regulations prohibit archers from being on the firing line in drives. Archers usually position themselves in elevated stands or portable “tree stands”. A bow shot can result in a missed shot, an arrow passing through the animal, or an arrow remaining lodged in the animal. In the latter case, a tracking dog is used. Reloading a bow takes time, so archers typically have only one shot per animal. A training program with a certificate, International Bowhunter Education Program (IBEP), coming from the USA, has been translated and was proposed to members of the Belgian Federation of Bowhunting (In Belgium, there are three federations, one Walloon and one Flemish, in addition to the Belgian Federation.), but it currently holds no value for the administration. Bow hunting is a minority practice and even more so for wild boar hunting. Nevertheless, according to those who engage or are familiar with it, it is considered the most respectful method in terms of animal tranquillity and the peace of other forest users. It is believed to carry a specific ethical standard. These practitioners hypothesised that bow hunting causes less suffering due to the impact of projectiles and arrow wounds, provided that the shot is effective. In the case of injury, proponents argued that the impact on animals is reduced. However, the large-scale adoption of this practice is contingent upon one subissue: the lack of certification, given the skills and qualifications required for effective practice. Nonetheless, it is important to emphasise that, at present, this practice does not have a certification or specific official training. Additionally, its scope is very limited as a solution for regulating populations at high densities like those of the wild boar, which is a concern.

#### 3.1.3. Searching for Wounded Wild Boar

In the Walloon region, searching for injured game, both small and large, is mandatory to reduce animal suffering given the ethical concerns. In driven hunts with dogs and trackers, about 15–20% (RSHCB 2023) (This number, which comes from the hunting license revision manual and is reissued every year, has been extensively discussed by some actors, some of whom pointed out its outdated nature and that it should be updated. During the meetings with the working group, it was decided to refer to the injured animals in this way: “The number of injured animals is difficult to estimate but involves several thousand individuals per year. Currently, ABUCS is not able to search for all these animals due to a lack of adequate resources. In 2022, the average success rate of these searches was 60% for all game combined.” [70]) of the animals are estimated as wounded. The Belgian Association of Blood Dog Users (ABUCS) is the only association in Wallonia dedicated to finding wounded game. Founded in 1985 [71], it follows German traditions and organises aptitude tests for dogs, training sessions, and awareness initiatives. A call centre coordinates searches, and, while services are voluntary, a small compensation for travel is customary. For small game and young wild boar, searches start immediately after the hunt, whereas for large game and big wild boar, a few hours’ delay is observed to avoid chasing a hot animal, which might flee or injure the dogs. Even so, some now argue for immediate searches.

Handlers of blood dogs recommend hunters to observe the animal’s reaction at the shot to assist them. When hunting is finished, hunters mark the shooting spot (for example, with a handkerchief) and limit their own checks to 50 m to avoid flushing the injured animal. They call a blood dog handler (sometimes already present and ready to intervene in big hunts). The handler checks for injury signs and, if positive, begins the search with their dog. The most--used dog breeds are Bavarian and Hanoverian scent hounds, but others like dachshunds can also be trained. The search may result in finding the animal dead or following it for kilometres. If the animal is alive and threatening, the handler kills it with a knife or a full metal jacket bullet, taking care not to injure the dog or themselves. Some stakeholders admitted to us that certain hunters might not contact a blood dog handler, thinking the animal was not hit or being reluctant to admit a bad shot. They also admitted that trophy animals are more often searched for than small wild boars.

Indeed, on the field, a proportion of shot animals do not succumb immediately to their injuries. Some escape and survive at the cost of suffering and varying degrees of mutilation, while others linger in agony for several hours after being wounded. Although the effectiveness of direct killing practices can be improved to limit these situations, a complementary issue is the search for wounded wild boar to end the animals’ suffering when necessary. Our investigation revealed several subissues; for the people we met, the challenge is to ensure that each shot fired undergoes a shooting check and, if necessary, a search if the initial result is positive. For them, it is also important to establish as a norm that hunters must systematically call for a search team. According to some interviewees, the use of blood-tracking dogs is increasingly sought, indicating a better awareness and response from hunters. On the ground, searches, when initiated, prove to be complex, and their outcomes uncertain.

Our survey revealed that the role of searching for wounded wild boar varies across hunting practices and carries both symbolic and practical significance. Success in tracking wounded animals is closely tied to the collaboration among hunters, particularly the accuracy of the shooter’s indications and the establishment of clear markers immediately after the shot. Properly marking the anschuss (the location where the animal was shot) is crucial, and some suggest that reinforcing best practices and ensuring all equipment is checked before hunting could improve search outcomes. The value of the animal also influences the search effort; high-value game like a stag with trophy potential is prioritised, while smaller wild boars, which may not be essential to the hunting plan, might not be pursued as diligently. A part of our interviewees met during this survey believed that too many shots are fired and animals are wounded without subsequent searches being conducted, mostly for wild boar. They advocated for strengthening the search framework. It is also noteworthy that the search for wounded wild boar is currently not subsidised by the Walloon region. Awareness of the issue of wounded game begins during hunting training and preparation courses. According to ABUCS, the ideal scenario would be for every hunt to have a qualified team to conduct searches. These interventions are necessary regardless of the hunting method: driven hunts, tracking and ambush, individual hunting, or road accidents. Overall, the search for wounded wild boar reflects the symbolic importance within the hunting community and is shaped by both practical collaboration and the value assigned to the animal. The difficulty of the search, the lack of official certification, and the highly specific skills required were highlighted as issues by stakeholders. These skills are generally passed down within the community. In Belgium, tests are organised by the associations themselves, which offer training courses and evaluations for both the dogs and their handlers. ABUCS and its handlers are said to have an obligation to achieve results without an obligation to provide means, which poses a problem in carrying out the numerous searches conducted by its members. In the field of search and rescue, a team consisting of a handler and a dog without an aptitude certificate is referred to as a “pirate handler”. Some search actors questioned whether, in the absence of enough handlers to perform all searches and prevent animal suffering, these pirate handlers should be legitimised. Nevertheless, how can one ensure the competency of such handlers if no training or tests have been conducted?

In terms of the well-being of the wounded animal, some participants emphasised the need to start the search as soon as possible. However, this search can be riskier for the dog. More broadly, when balancing effectiveness and safety, most field actors prioritised their own safety and that of their dogs. There are even instances where handlers may halt the search if there is a clear danger to the dog. According to the representative of the ABUCS association, on average, one dog is injured each year in the Walloon region. Most of these injuries result from encounters with wounded wild boars, which tend to seek refuge in thick cover.

#### 3.1.4. Putting to Death the Wounded Wild Boar

There was an ongoing debate among participants about the concrete methods of killing injured or dying animals. Given the risks to people, to the dogs accompanying them, and to avoid further suffering for the animal, stakeholders were uncertain about the best approach. Regulations (The legislation provides that: ‘The finishing of wounded big game is done with a bullet (…) By derogation from Article 6, it is however permitted (…) to use a knife to finish off a wounded big game animal.” [70]) in this area were sometimes criticised as unclear and sometimes questioned. First, in the context of searching for wounded game, an animal is killed with a blade if the beast is on the ground or with a firearm if the animal is still alive and cannot be approached. During a driven hunt, a wounded animal is killed with a firearm by a hunter on the shooting line and with a blade inside the beaters’ enclosure by beaters. Unlike in France, beaters cannot carry firearms during the drive. Properly finishing off an animal can be quite delicate, and accurately knowing where to place a knife to reach the heart, especially when the animal is in motion, is not always straightforward. This sometimes necessitates multiple attempts until the animal is completely dead. Some hunters prefer throat-slitting for a quicker kill. This raises again the issue of the knowledge or training of those who are tasked with killing animals in these circumstances (This operation can be carried out by a tracker, for example, and it is not mandatory to hold a hunting license to do so. The law does not specify the circumstances of the derogation nor the definition of a ‘knife,’ nor the precise procedures. A tracker can benefit from training organised by the RSHCB, which includes the methods of finishing, but this training is not mandatory [70]). Here, the involvement of dogs was both a support and a source of new challenges for the contributors.

#### 3.1.5. Cross-Cutting Issues Across Hunting Practices

We saw that each major hunting modality presents its own challenges in minimising harm to animal welfare. Even so, there were also cross-cutting issues that apply across different practices, which we address here.

Reducing Preventable Injuries and Suffering by Improving Technique Efficiency

There was consensus among the participants we interviewed (consensus was also reached on this issue within the working group subsequent to the investigation) that there is a direct link between the effectiveness of killing techniques and the reduction in harm to animal welfare. Among the technical aspects, they highlighted the choice of weapons and calibres, attention to shooting conditions, and the use of dogs.

Shooting and Conditions

The precise shot placement was crucial for our actors, especially with the shared goal of making each shot count. Achieving a perfectly placed shot on the animal results in a quick death with minimal suffering and avoids the scenario of a wounded animal escaping. To achieve such precision, beyond having the right equipment, excellent shooting conditions are necessary for the hunter, such as when the animal is stationary, calm, and well positioned. Our survey revealed that precision shooting is considered as feasible only at reasonable distances; the farther the animal, the more critical it is to be in the correct habitat, with the right equipment, and under the best conditions. Nevertheless, in many cases, shooting conditions are not ideal. In driven hunts, shooting must occur at shorter distances to potentially achieve a quality shot. Conditions can be improved by modifying the environment, such as increasing the size of the driving areas or enhancing shooting platforms for more comfortable ambushes. To encourage hunters to improve their shooting, some hunt managers now require hunters to retrieve their game or be taxed if the meat is completely ruined by a poor shot. Interviewed stakeholders in the hunting world argued that the situation is improving in terms of shooting effectiveness, partly due to advancements in techniques. Despite the general improvements in techniques and effectiveness noted in our survey, many contributors emphasised that too many animals are either mortally injured or mutilated.

Use of Tracking Dogs

One issue raised during our discussions was the use of tracking dogs and their overly aggressive behaviour, some of them having a very ‘biting’ character. Indeed, suffering and injury can result from interactions between a pack of dogs and the wild boar they chase, especially when the animal is small, such as piglets. Greater attention should be given to the training of these dogs and their breeding to reduce such incidents and their negative impacts on both wild boars and dogs according to our actors.

Establishing a Typology of Injuries

According to the actors we interviewed, it is essential to establish and consider the typology of injuries to assess their impacts on wild boars. Certain types of injuries almost invariably make it impossible to locate the injured animals; their carcass may be found later by chance by a forest ranger or another person roaming the woods. This raises significant issues regarding the suffering and living conditions of these animals following such injuries, as explained by a wildlife veterinarian who conducted autopsies on the game found dead in the forest.

Reducing Harm and Suffering by Enhancing Training and Skills of Field Practitioners

Beyond the preferred techniques and major hunting methods, a crucial issue for the stakeholders was the recognition and development of the skills of field practitioners. The practical examination for a hunting license aims to ensure that the hunter is proficient with their weapon, but it does not specifically train them to kill animals in the most effective way. This objective is somewhat implicit. One issue raised was the lack of verification of hunters’ skills beyond obtaining the license, particularly concerning shooting proficiency. Moreover, while the hunting permit is uniform, different hunting methods require different skills. Driven hunting, which demands the most shooting expertise, could be reserved for excellent markspeople, while stalking is more accessible from that point of view. In particular, for shooting skills, beyond initial training, the interviewed contributors advocated for regular practice and ongoing training to periodically refresh or improve skills. On the ground, a shared observation was that skills vary significantly from one practitioner to another: beyond the common hunting license, it is up to each hunter to refine their practice. Practising shooting requires having a nearby shooting range and attending it before the hunting season begins.

Ongoing training opens the door to validating these skills throughout the practitioner’s career. Beyond practical aspects, some participants also favoured more theoretical training or awareness-raising on certain topics to continually educate hunters. The hunting community members we interviewed expressed a desire for more training while noting that Walloon hunters already invest significantly in their own training compared to neighbouring countries. For instance, the Royal Saint-Hubert Club of Belgium (RSHC) (Largest hunting union in Belgium) has made investments to provide its members with modern shooting training equipment.

Amplified Issues Due to the Number of Animals Concerned

To introduce this issue, it is necessary to explain the dynamics of wild boar population densification at play in Belgium. Due to the dynamics of hunting leases and increasing hunting lease prices, practices have emerged to ensure a high and consistent number of game animals. Even so, not all hunts participate in these practices, and many hunters criticise them. The aim here is to explain these practices without pointing out specific responsible parties. We first explained the hunting methods to provide context for understanding these preparatory practices and their implications.

Three main practices are used to increase or stabilise wild boar populations in a given area, primarily targeting wild boars, as they best withstand hunting pressure while maintaining a healthy growth rate. The first one is feeding; this practice, strongly encouraged in the 1980s, involves two types. Supplementary feeding, aimed at helping game survive harsh winters, is typically used for deer, roe deer, fallow deer, and mouflons; the distraction feeding is intended to divert wild boars from cultivated fields to prevent damage for which hunters would be responsible. Both methods are often repurposed to increase wild boar populations for hunting purposes. Feeding concentrates to wild boar populations leads to weight gain, which in turn boosts reproduction since wild boars reproduce based on weight rather than age. “Baiting” is a related practice used during hunting season to keep wild boar groups in hunting areas. The second densification technique is reserving females; during hunts, female wild boars are often spared to allow for faster population growth. The last technique is illegal, as it involves the clandestine importation of live wild boars to be released into forests. Genetic analyses of the Walloon wild boar population support the theory of foreign introductions, showing diverse origins across Europe [72]. However, these results may be explained by the fact that importing wild boars was legal until 1994. What emerged from our field survey and meetings with actors was an acknowledgment of excess that pervades all hunting practices. This excess was thought to be the source of much suffering for wild animals. Wild boars are “artificially inflated” to meet the “always more” demand from some of those in the hunting world. For some participants, if the goal is to reduce animal suffering in killing practices, it seemed evident that reducing the occurrence of these practices by not promoting the overpopulation of these animals would be beneficial.

Minimising Animal Stress, Promoting Quietness

Several stakeholders advocated for a “regulation” approach to hunting, which involves large-scale and regular killing activities in the natural environment. At the same time, they argued that these activities and the associated techniques/practices should be as discreet as possible to preserve animal tranquillity. The question arose: how can one be both more discreet and more effective? This required the development of new tools and methods according to the participants, which are still largely unexplored and need to be debated to anticipate their broader impacts. It is also worth noting that hunters are not the only ones disrupting wild boar and other forest animals; domestic animals and their human companions also contribute to this disturbance, according to our actors.

Complementarity of Practices

Throughout the discussion of the issues related to hunting practices, our actors pointed out that it was not solely the practice itself that is problematic but often its context of use. For them, adjustments, stricter frameworks, and clarifications could make these practices less impactful on animal welfare. Indeed, in practice, different hunting modalities appear to complement each other to achieve current objectives for wild boar management: the complexity of the terrain, the diversity of local situations, and the range of prey justify a mosaic of practices. Some interviewees did not hesitate to explain how certain practices should coexist.

### 3.2. Knowledge Issues

#### 3.2.1. Lack of Scientific Knowledge to Establish Objectives and Indicators in Terms of Wild Animal Welfare

According to our survey, currently, in Wallonia, there is a lack of precise data to objectively measure the impact of hunting practices on the stress levels of wild animals. There have been no specific studies conducted by the administration or academics in Wallonia that focus on the welfare of wild animals or the factors that limit this welfare. Although the administration monitors the movements and activity rhythms of certain animals, such as wild boars, using tools like camera traps and GPS collars, these efforts are insufficient to establish concrete links between stress and hunting practices according to our actors.

Theoretically, it is possible to link stress levels to activity and hunting methods, which could identify territories and periods where animals are most stressed. However, implementing such studies is complex and costly due to the difficulty of collecting samples and the limited access to analysis laboratories. A crucial issue is defining what constitutes normal or acceptable stress for a wild animal to objectively identify stress-related harm. This definition can vary by species, population, or individual, necessitating preliminary reflection to establish priorities. The stakeholders interviewed relied on European scientific dynamics and international research projects to compensate for the lack of Belgian and Walloon studies. Despite some academic work, the deficit of local studies on wild animal welfare and their killing remains a significant obstacle.

Studies are ongoing to evaluate the effect of hunting methods on animals. A recent German study [52] measured cortisol levels (the stress hormone) in wild boars killed during drives, revealing increases in cortisol, but with significant variations depending on age, sex, and pregnancy status. The researchers concluded that there is considerable variability due to individuality, the duration of the chase, specific death conditions, chronic stress, microclimate, habitat structure, and hunting intensity. For a more precise evaluation, they suggested collecting samples throughout the year, on a larger scale, in different territories, and subjected to various hunting methods. That study demonstrated that precisely quantifying the impact of hunting methods on the game is complex due to the numerous factors involved, with each individual reacting differently to stressful situations. Beyond the hunting method and its frequency, the impact of the bullet on the animal should be studied much more extensively.

#### 3.2.2. Knowledge and Field Observations Underappreciated

This issue pertains to statistics and ultimately knowing how many animals there are and how many are killed. Regarding wild boars, there has been special effort since the African Swine Fever (ASF) crisis to obtain information. Nevertheless, in some areas, which do not fall under the hunting councils, are less likely to report their wild boar harvests. However, the administration estimates that this represents only 5 to 10% of the total harvest in the Walloon territory. In the 2019–2020 hunting season, 36,286 wild boars were killed or found dead [73] (It should be noted that this high number also resulted from the efforts to combat ASF among wild boars that year).

The issue of the number of animals was extremely prominent throughout our investigation for some participants. It involves counting the number of individuals per species in a given territory, as well as the number hunted or culled, and how they were killed. According to our contributors, it was important to mention that counting animals for hunting quotas or conservation varies in precision, as some species are not closely monitored. This makes it hard to justify culling for “overpopulation” without accurate data. The lack of tracking for both live and hunted animals limits the understanding of the true scale, welfare impacts, and species’ responses to management efforts.

For wild boars, hunting collectives (federations, associations) or groups that track injured game establish their own statistics according to their methods and objectives. Unfortunately, these statistics are not always aggregated or cross-referenced. The Walloon administration struggles to collect all field data other than mandatory shooting records, such as the number of wild boars killed in hunts, due to the vast number of actors involved. Furthermore, these records lack specific indicators to assess animal welfare, such as the frequency of different types of injuries or formally documented stress situations. Some interviewees suggested mandatory reporting for hunters, who should be encouraged or required to better document their kills, field observations, practices, and the effects of these practices. However, those directly involved raised concerns about the resources needed for such large-scale work, despite the emergence of online software like Geschasse (software to record search statistics or other hunting statistics) that facilitate data encoding and centralisation. Similarly, the idea of appointing observers for hunts was suggested.

### 3.3. Ethical Issues and Knowledge Issues

#### 3.3.1. Preserving the Wildness and Natural State of Wildlife

A shared observation among the stakeholders was that wildlife is not always in a state of well-being. Several quotes (excerpts from interviews conducted during the fall of 2022 with stakeholders from our investigation) illustrate this point:

- “There are particularly painful deaths in the animal world, in nature.”
- “Most deaths in nature are not immediate.”
- “In the wild, animals either die from disease, are killed by predators, or starve: whatever happens, there is suffering.”
- “The end of life for animals in the wild is, from our perspective, very harsh.”

Some actors explained to us that, in nature, animals have often evolved through natural selection as prey, existing with and driven by stress that encourages and accompanies escape behaviours. Several participants thus questioned if it is desirable to limit the stress of wild animals as we do for domestic animals. For several participants, wildness implies the autonomy and broad freedom of the animal, the ability to evolve spontaneously. Paradoxically, wilderness/wildness also refers to animals continuing to exhibit “typical” behaviours and ecology considered “ideal”. Our survey pointed out that the role of wildness in Wallonia likely deserves a broad debate to clarify the needs of wild animals and set objectives for minimising impacts on their welfare.

#### 3.3.2. A Claimed Hunting Ethics

During the interviews with stakeholders in the hunting community, the ethics of hunting was frequently mentioned. Based on our analysis and data, it emerged as a blend of several concerns regarding animals: caring for the population, giving the animal a chance (which can conflict with the goals of tranquillity or efficiency, described later), or conversely, leaving no chance at all (to avoid injuries).

Here, we outline the different elements raised by the contributors that revealed this ethic. Firstly, some elements contradict the hypothesis of purely regulatory hunting, devoid of any symbolic or identity-related relationship to the practice. Other elements highlight the boundary between preserving the wilderness (synonymous for most contributors with “autonomy of life” in a nature that can be cruel) and managing animal well-being. This well-being management seemed to be associated with (1) respecting the tranquillity of animals, (2) practices of domestication and care, or (3) seeking efficiency in the kill (ultimately synonymous with leaving no chance to the targeted animal, under the pretext of avoiding injuries and suffering).

Some interviewees incorporated into their hunting ethics the killing of wounded wild boars during the hunt without waiting until the end for safety reasons or the systematic search for all wounded game. The actors interviewed distinguished between the general ethics of hunting and the ethics in hunting. The latter refers to the actions taken, the “best practices”, in hunting adopted by hunters that translate the defended values into actions. For these contributors, a hunter who acts in accordance with the ethics of hunting formally identifies an animal before shooting. Conversely, shooting a female wild boar with piglets is considered by many hunters to be contrary to ethics.

Even so, it is important to note that our survey showed that hunting ethics are not uniform among all hunters, with actions categorised as ethical by some and not by others. There was no consensus on these issues or a single, shared ethics. In terms of understanding the ethics of hunting, our investigation raised questions rather than providing a definitive definition. It seems worthwhile to explore this aspect more broadly to use this ethic as a resource.

#### 3.3.3. Broad Recognition of Wild Animals as Sensitive Living Beings

After analysis of hunter training to obtain a hunting license, it would seem that at no point are wild animals considered from the perspective of their subjective and sensitive existence. Moreover, as previously noted, scientific studies on animal welfare and sensitivity have primarily focused on domestic and farm animals. Thus, we observed that beyond a general principle of common sense (and the fact that all vertebrates are both scientifically and legally recognised as sentient beings), there is a partially shared difficulty among field actors in fully recognising wild boars as beings endowed with sensitivity. In terms of detailed knowledge and the application of this knowledge in practice, these aspects were mostly denied until now.

It is therefore not surprising that among the participants interviewed, very few raised the consideration of sensitive living beings as a management issue. For most, when they assumed their role as managers, wild boars were seen as interchangeable, not as individual beings, or, if they were, they were confined to the categories reserved for their species. Several interviewees highlighted the uncertainties and lack of knowledge that would allow a nuanced understanding of the psychological distress and suffering experienced by wild boars. Despite the lack of objectification of this issue, they observed animal suffering in the field. Some participants, mainly hunters, did not hesitate to discuss the psychological or emotional suffering that hunted animals might experience, as well as the distress of those who survive after the death of their peers. While some interviewees acknowledged that wild boars “suffer like we do”, they seemed unable to connect this observation with the imperatives of wildlife management—whether it involves protected species, game, or even eradication efforts. This issue appeared in direct relation to the following point and the existence of a “polite” consensus (lack of public and ethical debate) around environmental management priorities: “population regulation” and “maintaining ecological processes” (discussed further from an environmental perspective). Integrating these goals with animal welfare was challenging for the stakeholders.

Nevertheless, for our interviewees, it was crucial to highlight that this difficulty arises in the absence of an explicit normative framework or knowledge dynamics that recognises and values these connections. Some environmental protection norms, on the contrary, encourage mistreatment when the rhetoric of war and destruction is employed. Terms such as “animals to be destroyed,” “pests”, and “invasive species” appear both regulatory and symbolically as gateways to abusive practices. The ethics of hunters and/or trappers and collective regulation then serve as barriers to such mistreatment. It is also important to note that these situations—of destruction and denial of the sensitivity of living beings—can potentially cause suffering for the humans involved and participating in these actions.

### 3.4. Ethical Issues Knowledge Issues

#### Balancing Ecological Priorities and Animal Welfare

In our survey, a major ecological issue championed by wildlife experts in the administration, associations, and academic circles was noted as significantly impacting the welfare of wild boars: the regulation of population density. Regarding the need to cull wild animals, there was a consensus on the justification related to population regulation. These stakeholders advocated for limiting, for example, wild boar for ecological, economic, or health reasons.

Some participants also pointed out that forest transformation necessitates reducing the density of certain animals, like wild boars. For many in the scientific and associative communities, and even some hunters, regulation seemed to be the only remaining valid justification for hunting. The current scientific consensus rules out the possibility of spontaneous population regulation. The need for regulation is compounded by a sense of urgency: immediate action is required to mitigate the damage caused by overpopulation amidst the biodiversity crisis. This urgency demands rapid and effective regulation and control measures to quickly reduce animal populations.

For our participants, it was crucial to recognise that the speed and scale of these regulatory/destruction operations, which pressure actors with limited resources, can significantly impact animal welfare. While game animals currently avoid these extreme forms of regulation/destruction, similar issues are evident in the exceptional destruction of wild boars through mass trapping and night shooting as part of the fight against ASF. Field contributors agreed that in such cases, it is no longer about hunting but about destruction, highlighting not only the gap between hunting and regulation but also the ethical dilemma surrounding these practices.

## 4. Discussion

In a context where “overpopulation” and “health crisis” led the authorities to classify wild boar as “to be destroyed”, hunters were prompted to reveal the practices and processes through which they established the existence of wild boars in order to differentiate themselves from the concept of mere destruction. These events opened the door to a broader debate, both outside and within the hunting world, on the welfare of these animals put to death.

We then followed the hypothesis that objectifying the welfare of wild boars was neither possible nor sufficient to ensure their management and that threats to this welfare were the result of relationships that can be described in their various facets. Another hypothesis was that in our societies, the existence of wild boars is largely established and inseparable from hunting practices, which vary widely and are themselves not uniform. This relationship is embedded in practices and trajectories that are situated, dynamic, and diverse (encompassing a variety of practices and situations in which this relationship unfolds). At the same time, these relationships are little recognised and utilised, while animal welfare governance has so far focused on domestic animals and a rather narrow view of the contributors and possible contributions to improving animal welfare. By doing so, we aimed to identify ways in which the existence of wild boars as sentient beings can play a more prominent role in the current and future debates on hunting and forest management.

Throughout our investigation, among the contributors, a range of avenues for reducing the impact on wild boar welfare during killing practices in the Walloon region emerged. These avenues would benefit from being considered with a certain degree of cross-disciplinary approach: In terms of practices, efforts should be made to enhance the effectiveness of killing by ensuring that it is as quick and direct as possible, minimising the animal’s suffering. Killing practices should also respect the animal’s adaptive capacity to the situation, fostering a sense of responsibility among hunters. This includes promoting selective practices, where careful consideration is given to which individuals to kill, along with a clear understanding of the compromises and ethical implications involved.

There is also a need to foster deeper reflections on the quality of interactions between hunters, wildlife, and the environment. To achieve this, training and skill development for practitioners should be enhanced, ensuring that they are well equipped to handle the complex situations they may encounter in the field. In terms of monitoring and knowledge production, it is important to develop a more comprehensive understanding of wildlife that goes beyond population dynamics and ecology. This should include a deeper appreciation of animals as sensitive and unpredictable beings, emphasising their welfare in management practices.

All the issues covered so far raised several questions about the actors’ ability to negotiate together to collectively manage this problem. Regarding organisation, there is a need to renew dialogue and governance bodies within the hunting community. Democratising hunting practices is essential to ensure that the world of hunting better reflects the values and expectations of the broader society. This can be achieved by promoting open and transparent dialogue between hunters, conservationists, and other stakeholders. Increased transparency in decision-making and management practices will help build trust and accountability within the community and beyond.

Through the descriptions presented in this document, it is evident that “hunting” cannot be regarded as a uniform practice nor can “hunters” be seen as members of a homogeneous community. In reality, practices that completely repel some hunters might be the very essence of hunting for others. Each participant in this microcosm, along with their auxiliaries, has different stakes and interests, each with their own way of experiencing or perpetuating hunting. This could be in a passionate, financial, recreational, familial, social, territorial, or traditional manner. These various perspectives inherent in the world of hunting are not necessarily perceptible or well represented in the statements of hunting representatives or within official bodies at the Walloon level. The spokespersons of the hunting world, whose positions are public, generally defend certain main issues and thereby overlook the diversity of positions among hunters. Many hunters felt that their particularities and vision of hunting were not represented.

Regarding the limitations of our investigation, several points can be highlighted. The results obtained may not be exhaustive or fully representative of all stakeholders in the hunting community. However, the participants interviewed, thanks to their extensive expertise on the issue, helped to raise important challenges that could be further explored from a quantitative perspective. It may also appear challenging to reconcile the knowledge produced here with that derived from veterinary medicine and wildlife management. Nevertheless, the issue of animal welfare requires a transdisciplinary approach. The results of this investigation can be discussed with our veterinary colleagues and serve as a basis for debate, while also providing a foundation for dialogue between experts and field actors. This approach does not replace a quantitative one but is intended to be complementary. Indeed, it also opens avenues for future quantitative studies and brings perspectives that are sometimes absent from traditional life sciences.

## 5. Conclusions

Our investigation among those involved in the killing of wild boars for the writing of this report indicated that there are many avenues for minimising harm to the welfare of wild boars in hunting practices. While it is unlikely that the welfare of each wild boar—whether in the context of hunting or outside of it—can be a realistic goal, this notion of welfare proved to be a good boundary object for debating different modes of human–animal relationships. Among the proposed actions, most involve the mobilisation of hunters but also depend on their collaboration with a variety of participants. Our results encourage the development of these collaborative spaces, which, at present, are very limited. However, it is important to highlight the innovative nature of our investigation and our involvement in this process: for the first time, the CWBEA based its work, and thus its approach to welfare, on the knowledge drawn from a social science methodology. This proves that beyond the care intended for wild boars, there is also an effort and a care to address the situation and the relationships between the stakeholders. To a greater extent, welfare research and its consideration in decision-making and wildlife management tend to encourage the health and well-being of humans, animals, and ecosystems like the One health approach claim.

Nevertheless, our results, like the literature review, show that much remains to be addressed in these areas to account for the sentient existence of the wild boar, which is sensitive and sentient in the sense of being capable of feeling and susceptible to suffering but also highly relational, with the ability to interact with humans. Additionally, they are sensitive and sentient in the sense of being problematic and controversial. The fascinating paradox here that was observed is this shift towards destruction during the ASF crisis that coincided with the emergence of concern for the welfare of wild boars and allowed these concerns to extend to traditional hunting practices.

The problem is that we are on a long trajectory, and this process is still ongoing. We wanted to highlight that this study is a phase of this process, an exploratory survey to prepare for the tasks of CWBEA working groups, without knowing its outcome and how to possibly integrate its results into political decision making. Nonetheless, we hope that this article is a contribution that outlines ways to ensure that the existence of wild boars as sentient beings, considered as gams, health threats, or pests, is more strongly considered in the current and future debates on hunting and forest management.

## Figures and Tables

**Table 1:** As part of our investigation, 22 key stakeholders ^1^ were interviewed between August and November 2022.

Status and Profile	Selection	Methods of Interviewing
An official from the administration, wildlife management expert	Person representing the department, knowledge, and/or acquaintance in another professional context and recommendation by other stakeholders	Remote interview via video call
Representative of an association for tracking wounded game	Search for this profile by the authors and people representing the department	In person at their residence
Veterinarian, hunter, and former collaborator of the European hunters’ association	Contacted the authors to participate in this study ^2^	In person at their residence
Representative of a hunting federation	Search for this profile by the authors, person representing the department and recommendation by other stakeholders	In person at their residence
Doctor of veterinary sciences, hunter, and bow hunter	Recommendation by other stakeholders	Remote interview via video call
Doctor of veterinary medicine, scientific advisor to a hunters’ federation	Search for this profile by the authors and people representing the department	In person at their residence
Representative of a federation of bow hunters	Search for this profile by the authors and people representing the department	In person at their residence
Representative of an environmental association specialising in plain wildlife and its habitats	Search for this profile by the authors and people representing the department	Remote interview via video call
Biologist, professor at a university, representative of a collective of environmental associations	Search for this profile by the authors and people representing the department	Remote interview via video call
Practising veterinarian, big game hunter and hunting manager	Recommendation by other stakeholders	In person at their workplace
Practising veterinarian, small game hunter, and hunting manager	Recommendation by other stakeholders	Remote interview via video call
Chief of a DNF district	Search for this profile by the authors and recommendation by other stakeholders	In person at their workplace
Hunting trainer, big game hunter, hunting manager, and expert in culling	Recommendation by other stakeholders	In person at their residence
Professor of veterinary medicine, specialist in the health and diseases of wild animals	Search for this profile by the authors, person representing the department and recommendation by other stakeholders	In person at their workplace
Former member of trapping unit (CIEI) and DNF officer	Search for this profile by the authors	In person at their workplace
DNF officer, big game hunter, and hunting manager	Search for this profile by the authors	In person at their workplace
Representative of an environmental association, specialist in destruction exemptions	Search for this profile by the authors, person representing the department, knowledge and/or acquaintance in another professional context	Remote interview via video call
Administrative officer, specialist in wildlife legislation	Search for this profile by the authors and people representing the department	Remote interview via video call
Lawyer, author of the first French Animal Law Code, head of a university diploma program in animal law	Search for this profile by the authors	Remote interview via video call
Lawyer, research on how legal norms structure relationships between human and nonhuman animals	Search for this profile by the authors	Remote interview via video call
Lawyer, specialist in international and European Union law, and animal welfare law	Search for this profile by the authors	Remote interview via video call

^1^ It should be noted that the list here includes all the individuals interviewed as part of the investigation in its entirety. This article focuses solely on the results related to wild boar hunting, so some interviews are not included here. ^2^ Some individuals contacted us themselves to participate in this study after hearing about it. We first assessed their profiles to determine if they were suitable for our investigation before agreeing to include them.

**Table 2:** The CATWOE grid [64].

C	Customers	The victims or beneficiaries of “T”
A	Actors	Those who would do “T”
T	Transformation process	The conversion of input to output
W	Weltanschauung	The worldview t makes this T meaningful in context
O	Owner	Those who could stop “T”
E	Environmental constraints	Elements outside the system which it takes as given

## Data Availability

The results of the complete survey can be found at this link: https://bienetreanimal.wallonie.be/files/documents/Avis%20CWBEA/Dossier%20scientifique%20%c3%a0%20destination%20du%20CWBEA%20-%20Animaux%20sauvages%20et%20mise%20%c3%a0%20mort%20-%202022%20(%20V2024%20Anonyme)%20Emond%2c%20Denayer.%20Uli%c3%a8ge.pdf. (accessed on 18 November 2024). The remaining data not used in this document are confidential or were not shared because they could have compromised the anonymity of the participants.

## Supplementary Materials

The following supporting information can be downloaded at: https://www.mdpi.com/article/10.3390/ani14233370/s1, Interview guide.
