# Supplementary material for: The Hunt for Kinder Practices: Minimising Harm to Wild Boar Welfare, Insights from a Qualitative Study in Wallonia (Belgium)
Source: Animals (Basel). 2024 Nov 22;14(23):3370. doi: 10.3390/ani14233370 (PMC11640469; doi:10.3390/ani14233370)
Supplement: Supplementary file 1 [file animals-14-03370-s001.zip › animals-3312292-supplementary.pdf]

animals

SUPPLEMENTARY FILE

MDPI

Article

The hunt for kinder practices : minimising harm to wild boar welfare, insights from a qualitative study in Wallonia (Belgium)

Pauline Emond and Dorothée Denayer.

Interview guide

Survey welfare and wildlife 2022

Team Introduction and Framework Reminder

Project Presentation:

- How are your practices and experiences related to the objectives of our investigation?
- What practices or instances of killing, in your view, negatively impact the welfare of wild animals? Can you describe them?

Questions Exploring Key Issues:

"Minimizing harm to the welfare of wild animals during their killing":

- What changes would this bring for you?
- What impacts would it have on you?
- How would you proceed?
- What do you stand to gain or lose if practices evolve?
- Who do we need to involve to make these changes possible? What resources, including technical ones, are required?
- In your opinion, who has the power to decide or improve the situation?

Additional Questions if Topics are Unaddressed:

- What is your stance on bow-and-arrow hunting?
- Are there resource persons we should meet (in France and Germany as well)?
- Do you know any resource persons specializing in the topics we just talk about?
